# Soil Moisture Determines Horizontal and Vertical Root Extension in the Perennial Grass *Lolium perenne* L. Growing in Karst Soil

**DOI:** 10.3389/fpls.2019.00629

**Published:** 2019-05-15

**Authors:** Jing Zhang, Jiamin Wang, Jinyi Chen, Haiyan Song, Suhui Li, Yajie Zhao, Jianping Tao, Jinchun Liu

**Affiliations:** Key Laboratory of Eco-environments in Three Gorges Reservoir Region (Ministry of Education), Chongqing Key Laboratory of Plant Ecology and Resources Research in Three Gorges Reservoir Region, School of Life Sciences, Southwest University, Chongqing, China

**Keywords:** root expansion, soil depth, drought, root surface area, root length density

## Abstract

Karst regions are characterized by heterogeneous soil habitats, with shallow wide soil (SW) on hilly slopes and deep narrow soil (DN) in rocky trenches. To make full use of limited water and nutrients, plants have therefore developed a number of root extension strategies. This study investigated the effect of soil moisture on horizontal root extension in SW and vertical root extension in DN by assessing root growth responses, biomass allocation, and root distribution. A full two-way factorial blocked design of soil dimensions by water availability was followed. The perennial grass *Lolium perenne* L. was grown in SW and DN under high (W100%), moderate (W50%), and low (W30%) water availability, respectively. The main results were as follows: (1) The total biomass of *L. perenne* was not influenced either by soil habitat or by water application. Root length, root surface area, root biomass and root to shoot ratio all decreased with decreasing water application in SW, but not in DN soil. (2) With decreasing water application, the cumulative percentage of root length, root surface area and root biomass in 4 rings from the center out to 12 cm of SW soil showed a trend of W50% > W30% > W100% in SW, however, the cumulative percentage of root biomass in 4 layers from the surface to a depth of 36 cm was not significantly different between different water treatments in DN. (3) Under all three water treatments, specific root length showed an increase but root length density showed a decreasing trend from the center outward in SW soil or from the surface to bottom in DN soil. Overall, these results suggest that in SW habitat, soil moisture determines horizontal expansion of the roots in *L. perenne*, although the overall expansion ability was limited in severe drought. However, due to the relatively strong water retention ability, soil moisture changes were less obvious in DN, resulting in no significant vertical extension of the root system. The root response of *L. perenne* helps our understanding of how herbaceous plants can adjust their belowground morphology to support their growth in harsh karst soil environments.

## Introduction

The root system supports plant growth by absorbing and transporting water and nutrients (Xu et al., 2017). It has therefore become an important focus of studies aimed at ecosystem ecology and global climate change research (Copley, 2000; Morgan, 2002) and is likely to still increase in importance (He et al., 2004).

In terrestrial ecosystems, the root system can change its morphological structure in response to the surrounding soil environment (Ren et al., 2011). Such strong plasticity is an important survival strategy in certain environments (Wang et al., 2017). Root morphological plasticity is also closely related to plant growth strategies and the ability to exploit environmental resources (Wang et al., 2017). Any change in plasticity will therefore have an effect on plant growth (Wang and Mou, 2012), playing an important role in environmental adaptation (Zhang and Xu, 2010). For example, morphological plasticity of the root system determines the position and extension ability of the roots (Malamy, 2005). Moreover, it also affects the utilization of belowground resources (Zhang et al., 2005) as well as the efficiency of water and nutrient uptake, and therefore, the plants’ adaptability (Ji and Yang, 2011; Ren et al., 2011; Wang et al., 2017). Thus, horizontal and vertical distribution of the roots is the basic manifestation of a plant’s ability to gain water and nutrition (Zhang et al., 2005).

Root expansion ability is closely related to environmental conditions, and is largely influenced by soil and climate factors. Soil depth and soil permeability are important factors affecting expansion of the root system (Yang, 2012), with soil depth affecting root growth and distribution in terms of space, water availability and nutrition (Mcconnaughay and Bazzaz, 1991; Hess and De Kroon, 2007; Felten and Schmid, 2008; Li et al., 2018). Studies also suggest an increase in water and nutrient stores in deep soil, offering wider potential for root spread and giving deep-soil plants the opportunity to increase their root distribution range and expansion (Gao et al., 2013). However, an increase in soil depth also has a negative effect on soil structure and permeability (Geng, 2013).

Deep soil tends to be compacted and contain less oxygen, is not easy for roots to obtain water and nutrients from it. Especially under drought conditions, the roots have to overcome greater friction when they extend downward into very deep soil (Liang and Chen, 1995). In contrast, in shallow soil, with limited vertical rooting space, roots mainly take up water and nutrients from the upper layer, resulting in a well-developed horizontal root system. However, due to increased evaporation and a poorer ability to hold water, drought stress is much more common in shallow soil; thereby the horizontal expansion of the roots will also be restricted.

Karst regions represent a unique ecological system consisting of heterogeneous soil habitats. Due to the influence of carbonate parent rock, the soil is dominated by limestone, which is characterized by slow soil formation, shallow soil layers, broken terrain and bare bedrock. Such habitats therefore consist of shallow, discontinuous soil on hilly slopes and deep continuous soil in rocky trenches and crevices (Yang, 1990; Li et al., 2006). Due to the spatial heterogeneity of soil distribution in such regions, the moisture distribution is also highly heterogeneous (Guo et al., 2011). In shallow karst soil, the water storage capacity is usually poor, due to a lack of soil volume, poor soil quality and strong permeability (Zhou et al., 2002). In such areas, soil droughts are common. In contrast, deep soil regions are able to maintain a relatively good soil structure and moisture level due to their strong water storage capacity. However, a decrease in rainfall amount and rainfall frequency due to global climate change could result in an increase in the frequency and intensity of karst droughts, leading to further deterioration of karst habitats, with serious negative effects on plant growth and poor prospects of vegetation restoration.

To make full use of water and nutrient resources, plants in karst environments have developed a number of root expansion strategies. In shallow soil habitats, such as stoney soil and depressions with sporadic soil distribution, plant roots tend to extend horizontally, rather than vertically (Luo et al., 2010), allowing them to access moisture from an unconstrained area (Nie, 2011). However, as mentioned above, plants are subjected to more severe drought stress in these shallow soil habitats, and must therefore exploit a large area of soil underneath the rocky surface (Poot and Lambers, 2008). In contrast, plants growing in deep soil habitats such as rocky trenches and crevices show greater longitudinal root extension. This ability is further promoted by the reduction in soil moisture in such habitats (Bai et al., 2001), increasing root length, root surface area and root volume to optimize access to water in deeper soil layers (Ding et al., 2013a,b). Moreover, by accessing water stored in rock cracks and fissures, karst plants are better able to tolerate drought stress conditions (Schwinning, 2010). However, while deep-soil karst habitats maintain a degree of soil structure and certain moisture level, drought remains a problem. Such soil is therefore low in available nutrients, susceptible to compaction, and thereby poor aeration. Thus, deep vertical root extension also faces challenges. Understanding the limitations of longitudinal root extension under insufficient water and nutrient conditions is therefore important.

The expansion strategy of root systems in karst regions is therefore affected not only by soil depth, but also by additional lack of water. Thus, we hypothesized that in shallow soil habitats, horizontal expansion will be promoted under moderate drought conditions; however, under severe drought, the range of horizontal extension is expected to be limited. In contrast, in deep soil habitats, vertical expansion is expected to be promoted under drought conditions, with a decrease in expansion under severe drought.

Research on vegetation restoration and reconstruction suggests that the soil and water conservation capacity of artificial grassland in karst areas is higher than that of any other vegetation type (Yang et al., 2017). Grasses are characterized by rapid growth and sensitivity to environmental change. Promotion of grassland areas and grassland animal husbandry could therefore act as an effective way of addressing ecological deterioration in karst regions (Li et al., 2011). To examine the above hypotheses, growth of *L. perenne*, a candidate species for karst vegetation restoration, was examined in shallow and wide vs. deep and narrow soil reflecting karst habitats. Three water treatments were also applied based on precipitation levels in recent years, with the aim of exploring root extension strategies, respectively, in shallow wide soil (SW) and deep narrow soil (DN) with the reduced water supply. The findings will provide important information for restoration of karst ecosystems.

## Materials and Methods

### Test Materials

*Lolium perenne*, a perennial Poaceae grass with fibrous root system, was used as the test plant. The seeds were purchased in Chongqing Zhongye Industry Co., Ltd. Yellow limestone soil from a karst region on Zhongliang Mountain, Chongqing, China, was used as the test soil. The soil had a pH of 7.4 ± 0.14, organic matter content of 0.34 ± 0.02%, total nitrogen content of 0.28 ± 0.03 g/kg, total phosphorus content of 0.39 ± 0.02 g/kg, total potassium content of 23.7 ± 3.22 g/kg, field water holding capacity (FC) of 39.8 ± 2.23%, and soil bulk density of approximately 0.013 g/cm^3^.

### Experimental Design

**Figure 1:**
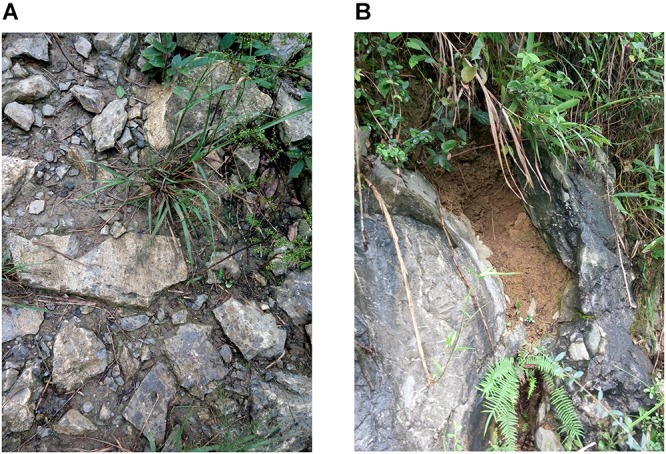
Two typical habitats in a karst region in Zhongliang Mountain, Chongqing, China. **(A)** Shallow and wide soil habitat on hill slope. **(B)** Deep and narrow soil habitat in rocky trench and crevices.

The experiments were carried out at an altitude of 225 m in the experimental garden of Southwest University Ecological Park (29°49′N, 106°25′E), Chongqing, which has a subtropical monsoon climate. To simulate the karst soil habitats, that is, shallow and wide vs. deep and narrow habitat (Figure 1), two soil profiles were examined, shallow wide (SW) soil with a length, width and height of 30, 30, and 5 cm, respectively, and deep narrow (DN) soil of 5, 5, and 45 cm, respectively. Soil profiles were created in containers of the above specifications using the same volume of soil. Containers had holes in the bottom to allow drainage. Each container was equipped with 5 kg of yellow limestone soil, which had been air-dried and milled.

Seeds of *L. perenne* were sown in a seedling tray on July 9, 2017. After 15 days, seedlings showing consistent growth were then transplanted in the center of a designated container. The soil was kept moist to ensure survival and growth. On September 21, 2017, 48 seedlings of approximately 40 cm in height were selected for water treatment. Water treatments were based on the precipitation patterns in Chongqing in the past 30 years (1981–2011). Using the daily average (2.63 L/m^2^) from September to November, average daily rainfall was calculated for each container type based on the bottom area. Accordingly, the following three water treatments were established: control, normal water conditions (W100%), mild drought (50% reduction, W50%) and severe drought (70% reduction, W30%). Water was applied every 3 days. The watering treatments continued for 60 days. Therefore, the full factorial, randomized block design included two factors namely soil depth (SW vs. DN) and water availability (W100%, W50%, W30%). There were six treatments in total with four replicates in each treatment. Details of each water treatment are shown in Table 1.

**Table 1:** Average daily precipitation, interval days and watering of each treatment.

	Average daily precipitation (L/m^2^)	Interval days (d)	W100% (mL/3d)	W50% (mL/3d)	W30% (mL/3d)
SW	2.63	3	711	355.5	213.3
DN			78	39	23.4

*SW, shallow wide container; DN, deep narrow container. W100%, W50%, and W30%: high, moderate and low water availability, respectively*.

### Measurements and Calculations

**Figure 2:**
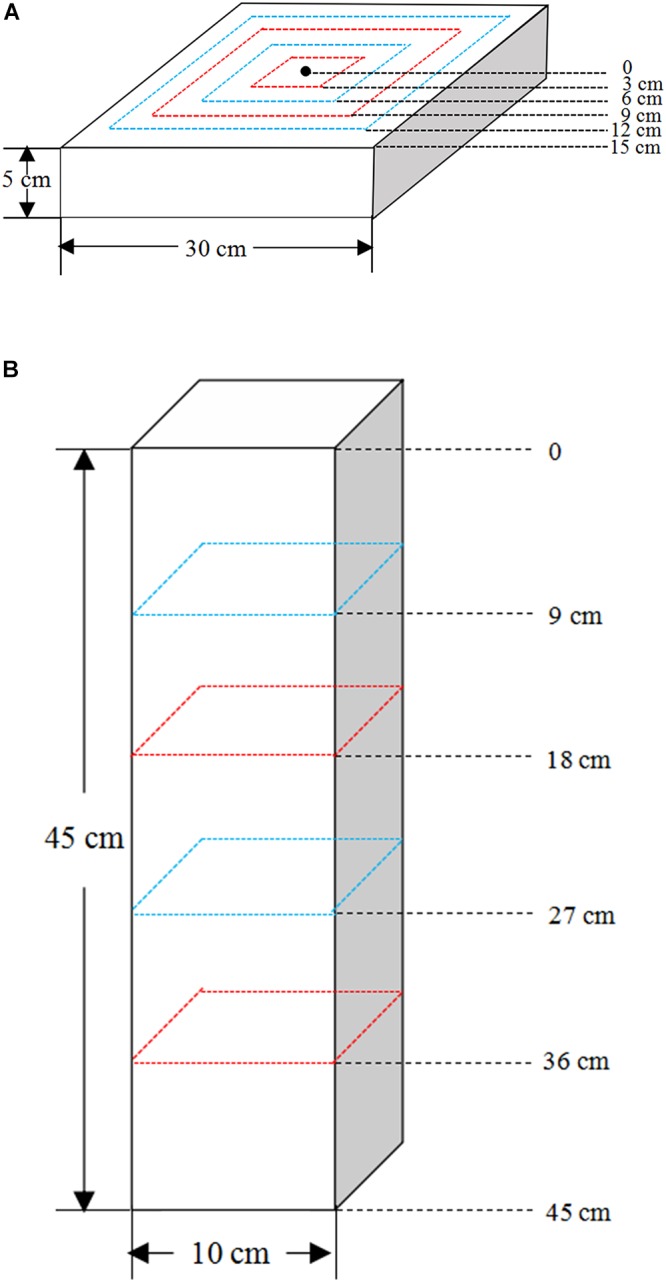
The root sampling design of **(A)** the shallow wide (SW) and **(B)** deep narrow (DN) containers.

All plants were harvested and separated into roots and shoots after 60 days of water treatment. The shoots of each plant were harvested, put into respective envelopes and dried at 60°C to a constant weight. Then, after cooling to room temperature in a desiccator, they were weighed to determine the aboveground biomass. The roots were harvested in a stratified manner. Firstly, soil in SW container was sampled at 3 cm intervals from the center to the outer rim of the container at 0–3, 3–6, 6–9, 9–12, and 12–15 cm, respectively. And soil in DN container was obtained at 9 cm intervals from the top to bottom at 0–9, 9–18, 18–27, 27–36, and 36–45 cm, respectively (Figure 2A,B). Thus, the soil of each container was divided into five rings or layers. Meanwhile, soil samples of approximately 5 g were collected from each soil ring or layer, respectively, and placed in a purpose-made aluminum box to be returned to the laboratory. The soil water content (SWC) of each ring or layer was measured by weighing. Secondly, the remaining soil together with the roots of each soil ring or layer was then placed in a plastic pot, soaked in water and strained through a 1 mm sieve. Finally, the roots were picked out by tweezers carefully and placed in a zip lock bag, then analyzed after thorough cleaning. The root images of each ring or layer were then obtained using a digital scanner (STD1600, Epson, United States), and the corresponding root length and surface area were analyzed using WinRhizo (Version 410B, Regent Instrument Inc., Canada). Then the roots were placed in respective envelopes and dried at 60°C to a constant weight. After cooling in a desiccator, they were weighed to determine the root biomass of each ring or layer. We calculated a number of indices describing the biomass allocation, spatial distribution of roots and moisture content within the soil. Calculations of these indices are shown in Table 2.

**Table 2:** Calculations of indices.

Indices	Calculations
Soil water content (SWC)	The average water content of each ring in SW soil or each layer in DN
Soil relative water content (SRWC)	SWC/field water holding capacity (FC) × 100%
The total length of the roots	The sum of root length of each ring or layer
The total surface area	The sum of root surface area of each ring or layer
The total biomass of the roots	The sum of root biomass of each ring or layer
Total biomass	Shoot biomass + total root biomass
Root shoot ratio	Total root biomass / shoot biomass
Root length density	Root length of each ring or layer / soil volume of each ring or layer
Specific root length	Root length of each ring or layer / root biomass of each ring or layer
Percentage of root length of each soil ring or layer	Root length of each soil ring or layer / total root length × 100%
Percentage of root surface area of each ring or layer	Root surface area of each ring or layer / total root surface area × 100%
Percentage of root biomass of each ring or layer	Root biomass of each ring or layer / total root biomass × 100%
Cumulative percentage of root length of 1-n ring or layer	Percentage of root length in 1st ring or layer + percentage of root length in 2nd ring or layer + percentage of root length in 3rd ring or layer +...+ n, (*n* = 2,3,4,5)
Cumulative percentage of root surface area of 1-n ring or layer	Percentage of root surface area in 1st ring or layer + percentage of root length in 2nd ring or layer + percentage of root length in 3rd ring or layer +...+ n, (*n* = 2,3,4,5)
Cumulative percentage of root biomass of 1-n ring or layer	Percentage of root biomass in 1st ring or layer + percentage of root length in 2nd ring or layer + percentage of root length in 3rd ring or layer +...+ n, (*n* = 2,3,4,5)

*First–fifth ring was 0–3, 3–6, 6–9, 9–12, and 12–15 cm, respectively, in SW, while 1st–5th layer was 0–9, 9–18, 18–27, 27–36, and 36–45 cm, respectively, in DN*.

### Data Analysis

The effects of soil space, water and their interactions on plant growth, and biomass accumulation and allocation were evaluated via Two-way ANOVA using SPSS 22.0. The effects of soil layer, water and their interactions on root distribution were also evaluated via Two-way ANOVA using SPSS 22.0. All figures were produced using Origin 2017.

## Results

### Soil Water Content

No significant differences in SWC were observed between the SW and DN containers under W100% water treatment. However, with a reduction in water application, the SWC decreased significantly under W50 and W30% treatment by 20 and 30% in SW and by 9 and 16% in DN, respectively (Table 3). Two-way ANOVA revealed highly significant effects of container and water treatment on SWC, and a significant container × water treatment interaction (Table 4).

**Table 3:** Effect of water treatment on the soil moisture content in each container type.

	SW		DN	
	Soil water content (%)	Soil relative water content	Soil water content (%)	Soil relative water content
W100%	20.24 ± 0.25a	51% FC	20.32 ± 0.77a	51% FC
W50%	16.26 ± 0.19b	41% FC	18.42 ± 0.45b	46% FC
W30%	7.37 ± 0.24c	19% FC	17.10 ± 0.33b	43% FC

*Lowercase letters indicate a significant difference between water treatments at *p* = 0.05. SW, shallow wide container; DN, deep narrow container. W100%, W50%, and W30%: high, moderate and low water availability, respectively. Values represent the mean ± SE*.

**Table 4:** Two-way ANOVA results of the effect of water treatment and container type (SW vs. DN; see Table 1) on the soil moisture content and root indices.

Index	*F*-value		
	Container	Water	Container × Water
Soil water content	7.40**	52.23***	16.78***
Soil relative water content	135.77***	188.60***	73.25***
Total biomass	0.70 ns	1.25 ns	0.97 ns
Root biomass	1.13 ns	6.75**	2.38 ns
Root shoot ratio	11.35**	7.19**	4.04*
Root length	1.44 ns	7.27**	2.70 ns
Root surface area	1.33 ns	8.03**	3.61*

^∗^*P < 0.05, ^∗∗^*P* < 0.01, ^∗∗∗^*P* < 0.001, ns, no significant difference at *p* = 0.05*.

Similarly, no significant differences in SRWC were observed between the SW and DN containers under W100% water treatment. Moreover, the moisture content of both containers was 51% of the FC, suggesting mild drought. However, with a reduction in water application, the SRWC under W50 and W30% treatment reached 41 and 19% FC in SW, suggesting moderate and severe drought, respectively. Meanwhile, in DN 46 and 43% FC were observed, respectively, suggesting moderate drought under each treatment (Table 3). Two-way ANOVA revealed highly significant effects of container and water treatment on SRWC, and a significant container × water treatment interaction (Table 4).

### Biomass Accumulation and Allocation

**Figure 3:**
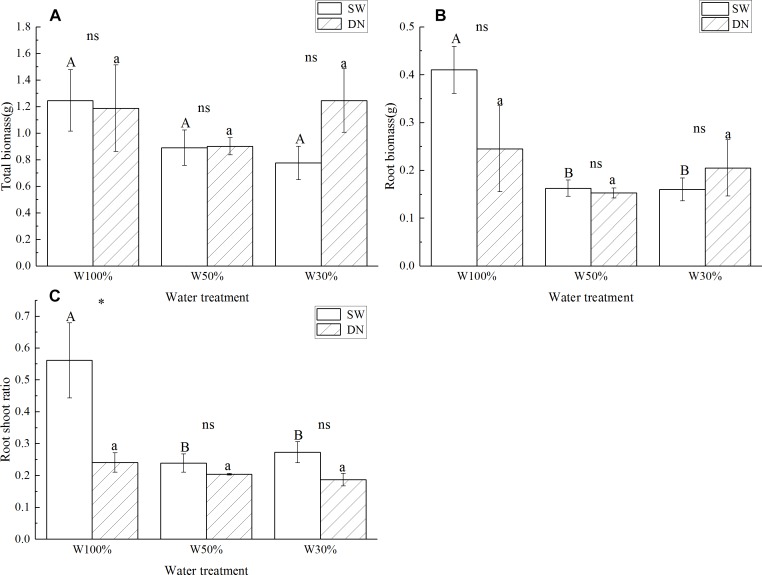
Effects of water treatment and soil type on **(A)** the total biomass, **(B)** the root biomass, and **(C)** the root to shoot ratio of *Lolium perenne*. Values represent the mean ± SE. W100%, W50%, and W30%: high, moderate and low water availability, respectively. SW, shallow wide container; DN, deep narrow container. Different capital letters indicate a significant difference between water treatments at 0.05 level in SW. Different lowercase letters indicate a significant difference between water treatments at 0.05 level in DN. ^∗^indicates a significant difference between different soil treatments under the same water treatment at 0.05 level, ns indicates no significant difference at the 0.05 level.

No significant differences in total biomass were observed between SW and DN in any water treatments. Meanwhile, total biomass did not change with decreasing water application from W100 to W30%, neither in SW nor in DN (Figure 3A and Table 4). There were also no significant differences in root biomass between SW and DN in any water treatments. However, a decrease was observed in SW when the water application decreased (Figure 3B). Two-way ANOVA revealed a highly significant effect of water treatment on root biomass, but no significant effect of container or container × water treatment interaction (Table 4). The root to shoot ratio was higher in SW than DN at W100%. With decreasing water application, a significant reduction in root to shoot ratio was observed in SW, but not in DN (Figure 3C). Two-way ANOVA revealed highly significant effects of container and water treatment on the root to shoot ratio, and a significant container × water treatment interaction (Table 4).

### Root Length and Root Surface Area

**Figure 4:**
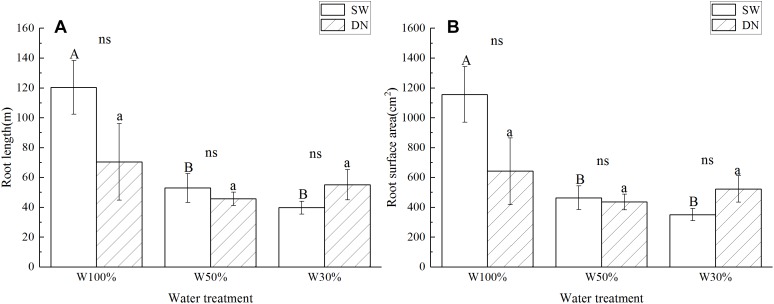
Effects of water treatment and soil (container) type on **(A)** the root length and **(B)** the root surface area of *L. perenne*. Values represent the mean ± SE. W100%, W50%, and W30%: high, moderate and low water availability, respectively. SW, shallow wide container; DN, deep narrow container. Different capital letters indicate a significant difference between water treatments at 0.05 level in SW. Different lowercase letters indicate a significant difference between water treatments at 0.05 level in DN. ns indicates no significant difference at 0.05 level.

Root length and root surface area showed the same trend as the root to shoot ratio (Figure 4A,B). Two-way ANOVA revealed no significant effect of container on root length or root surface area; however, a highly significant effect of water treatment was revealed. No significant container × water treatment interaction was observed in terms of root length; however, a significant effect on root surface area was revealed (Table 4).

### Root Distribution

**Figure 5:**
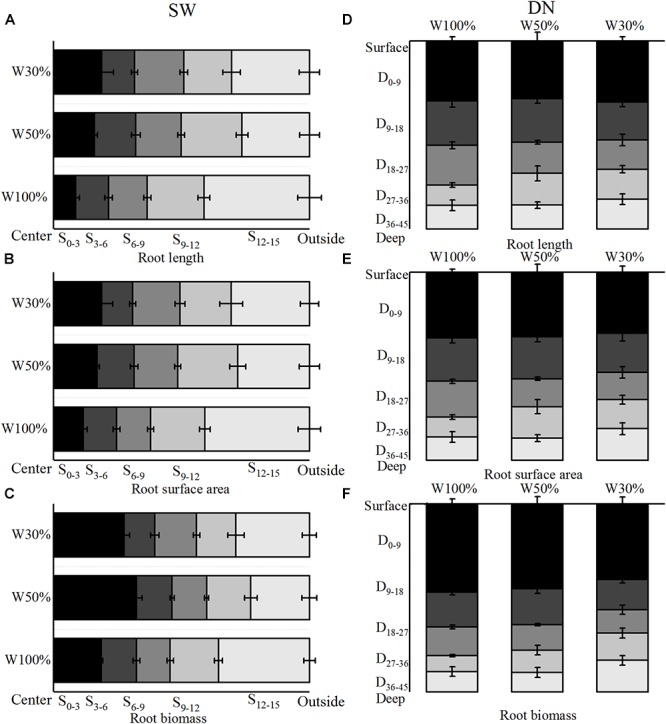
Effects of water treatment on **(A,D)** the distribution of root length, **(B,E)** the surface area, and **(C,F)** the biomass of *L. perenne* in the two soil (container) types. Values represent the mean ± SE. W100%, W50%, and W30%: high, moderate and low water availability, respectively. SW, shallow wide container; DN, deep narrow container. In SW, S_0–3_, S_3–6_, S_6–9_, S_9–12_, and S_12–15_ represent 0–3, 3–6, 6–9, 9- and 12–15 cm soil rings, respectively, while in DN, D_0–9_, D_9–18_, D_18–27_, D_27–36_, and D_36–45_ represent 0–9, 9–18,18–27, 27–36, and 36–45 cm soil layers, respectively.

With decreasing water application, the cumulative percentage of root length and root surface area in 4 rings from the center out to 12 cm of SW followed the order W50% > W30% > W100%. Meanwhile, the ratio of root length and root surface area in the fourth ring (9–12 cm) from the center followed the order W50% > W100% > W30% (Figure 5A,B). In contrast, the cumulative percentage of root length and the root surface area in 4 layers from the surface to 36 cm of depth in DN was not significantly different between W50 and W100%, while both had slightly higher values compared to W30%. However, the percentage of root length and root surface area in the deep soil layer of 27–36 cm (the fourth layer) followed a trend of W50% > W30% > W100% (Figure 5D,E). Two-way ANOVA revealed no significant effect of water on the distribution of root length or the root surface area; however, a highly significant effect of stratification was observed in both SW and DN. Moreover, a significant water treatment × stratification interaction was observed in SW, but not DN (Table 5).

**Table 5:** Two-way ANOVA of the effect of water treatment and soil stratification on the soil water content and root indices in each container type.

Container	Source of variation	*F*-value					
		Soil water content (%)	Root length (%)	Root surface area (%)	Root biomass (%)	Root length density (cm/cm^3^)	Specific root length (m/g)
SW	Water (W)	875.69***	0.12 ns	1.69 ns	0.00 ns	52.20***	14.60***
	Stratification (S)	1.62 ns	21.53***	21.06***	39.21***	44.98***	33.68***
	W × S	1.04 ns	2.48**	2.73*	6.07***	0.86 ns	1.41 ns
DN	Water (W)	7.02**	0.02 ns	0.01 ns	0.00 ns	1.49 ns	1.30 ns
	Stratification (S)	0.38 ns	19.64***	21.23***	98.96***	2.91*	9.78***
	W × S	0.24 ns	1.05 ns	0.97 ns	1.67 ns	0.46 ns	0.91 ns

^∗^*P < 0.05, ^∗∗^*P* < 0.01, ^∗∗∗^*P* < 0.001, ns, no significant difference at *p* = 0.05. SW, shallow wide container; DN, deep narrow container*.

With decreasing water application, the cumulative percentage of root biomass in 4 rings from the center out to 12 cm in SW followed a trend of W50% > W30% > W100% (Figure 5C). Meanwhile, in DN, the cumulative percentage of root biomass in 4 layers from the surface to a depth of 36 cm was not significantly different between W100 and W50%. However, in the deeper soil layer of 27–36 cm, the ratio of root biomass followed the order W30% > W50% > W100%, and nearly half of the biomass was distributed in the 0–9 cm layer under all three treatments (Figure 5F). Two-way ANOVA revealed no significant effect of water treatment on the distribution of root biomass; however, a significant effect of stratification was found in both SW and DN. Moreover, a highly significant water treatment × stratification interaction was observed in SW, but not DN (Table 5).

**Figure 6:**
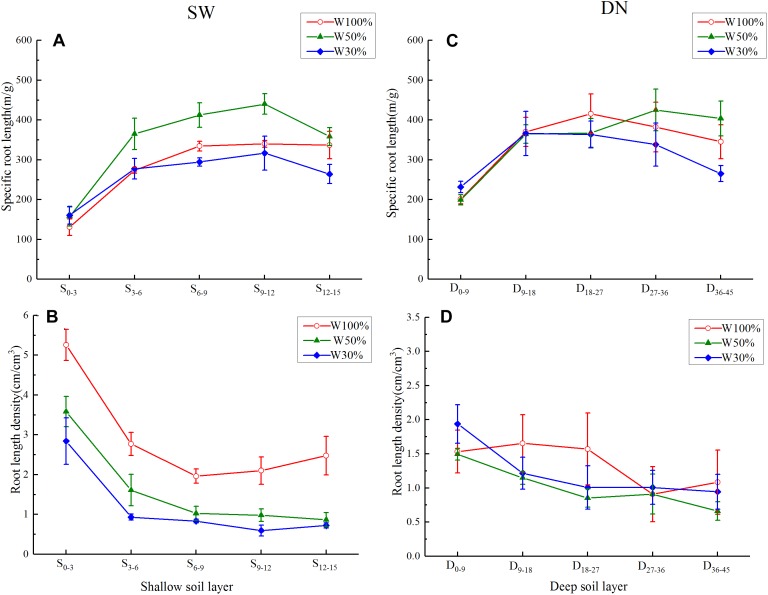
Effects of water treatment on **(A,C)** the specific root length and **(B,D)** the root length density of *L. perenne* in each soil layer in the two soil (container) types. Values represent the mean ± SE. W100, W50, and W30%: high, moderate and low water availability, respectively. SW, shallow wide container; DN, deep narrow container.

Under all three water treatments, a rapid and then slow increase (or a very slight decrease) in specific root length was observed from the center to the outer in SW, or from the surface to bottom in DN (Figure 6A,C). Overall, with decreasing water application, the specific root length of each soil ring in SW followed the order W50% > W100% > W30%. This was especially obvious in the 3–12 cm ring. Meanwhile, in DN, no significant differences between soil layers were observed under different water treatments, although a trend of W50% > W100% > W30% was observed in the deepest layer (36–45 cm) (Figure 6A,C). Two-way ANOVA revealed highly significant effects of water and stratification on the specific root length in SW, while in DN, a highly significant effect of stratification but no significant effect of water treatment was observed. No water treatment × stratification interaction was observed in either SW or DN (Table 5).

Under all three water treatments, the root length density showed a decreasing trend from the center outward in SW, or from the surface to bottom in DN. With decreasing water application, the root length density in each soil ring in SW decreased gradually, following the order W100% > W50% > W30%; however, there were no obvious differences between water treatments in DN (Figure 6B,D). Two-way ANOVA revealed highly significant effects of water and stratification in SW, while in DN a significant effect of stratification but no significant effect of water treatment was observed. No water treatment × stratification interaction was observed in either SW or DN (Table 5).

## Discussion

Soil is considered to be a complex environment affecting plant growth through the availability of water, nutrition and living space (Hess and De Kroon, 2007; Felten and Schmid, 2008). Many studies focusing on plants in karst areas have shown that limited resources including water and soil volume can inhibit the accumulation of plant biomass (Zhao et al., 2017). Inconsistent with these studies, the total biomass of *L. perenne* was not influenced either by the horizontal and vertical dimensions of available soil habitat or by water application. First, even though soil moisture decreased sharply with the decline of water application in shallow and wide (SW) soil, it did not cause a measurable space or resource limitation for *L. perenne*, a generally shallow-rooted plant. Second, the soil moisture under different treatments in deep narrow (DN) soil decreased only slightly in response to reduced water supply, probably mainly because of larger soil retention capacity due to the relatively lower level of evaporation (Zhang et al., 2013). The other finding was that *L. perenne* was very tolerant to drought. As a result, it could still keep the total biomass largely unaffected (Aronson et al., 1987) both in the two physically contrasting soil habitats and at different water applications.

However, we found that root length, root surface area, root biomass and root to shoot ratio were all greater at W100% in SW. Generally, drought will stimulate root growth over shoot growth to seek the water source deeper down in the soil (Schulze et al., 1996). However, *L. perenne* is a shallow-rooted plant and the soil in SW was very shallow. Other studies also indicate that, when facing dry patches, plants may develop smaller roots to enhance competitiveness because less carbon investment is required to maintain root structure and reduce root respiration (Berntson, 1994). Thus, the fact that *L. perenne* invested more biomass into roots at water-rich SW was possibly an adaptive response.

As hypothesized, *L. perenne* indeed developed horizontal expansion at moderate drought in SW, since, if excluding the marginal effect produced by the outer ring, the cumulative percentage of root length, root surface area and root biomass all showed a trend of W50% > W30% > W100% from the center out to 12 cm (Figure 5A,B); this could allow the roots to obtain more water (and thereby nutrition) for growth. Moreover, the specific root length was highest under W50% and the root length density in each SW ring decreased with decreasing water application (Figure 6A,B), also suggesting that water and nutrients were allocated mainly for horizontal root extension (Dang et al., 2012). However, the findings also suggested that the horizontal extension ability of the root system was limited when the soil water decreased to a severe drought level (W30%, 19% FC). From a resource economics perspective, it would indeed be non-adaptive for a plant to invest energy into a place without much opportunity for resources. Our results suggest that severe drought might encourage roots to grow downward in order to seek water and nutrients. However, if a mechanical obstacle was encountered during the growth process, downward extension of the root system would be reduced and the roots would be forced to grow mostly horizontally along the surface (Monshausen et al., 2009). This would explain the percentage of root allocation in the 12–15 cm soil ring in SW, which was relatively larger under W100% treatment, possibly due to extension of the root system along the surface of the impenetrable container bottom.

On the other hand, inconsistent with our hypothesis, vertical distribution of the root system of *L. perenne* into deep soil habitats was not observed. The soil moisture did not significantly decrease with decreasing water application in DN. Some studies have shown that water elevation and redistribution of the root system often exist in soil (Wang et al., 2013). Water in wet soil can be absorbed by the roots and then transported to roots in drier areas, some of which is then released into the dry soil around the rhizosphere under low transpiration conditions, mainly at night (Richards and Caldwell, 1987). It is generally believed that the direction of water elevation is from moist deep soil to dry shallow soil; however, studies by Burgess et al. (1998) and Smith et al. (1999) suggest that soil moisture could also be transported down to deeper soil layers by the root system. In our study, the soil moisture content of the subsequent layers did not differ significantly, which was possibly a result of water redistribution by the roots. As a result, it was not necessary for the root system to extend into the deep soil. As we mentioned, *L. perenne* has a shallow root system and somewhat succulent roots. The structure of the soil is also affected by an increase in thickness (Geng, 2013); this may have made it difficult for the shallow roots to extend downward, with soil depth acting as a major barrier to root growth beyond a certain depth.

However, in the deeper layer (27–36 cm) in DN, the ratio of root length and root surface area also had the trend of W50% > W30% > W100%, suggesting that if the soil moisture content was significantly different, vertical expansion of the roots would be expected in DN, but this expansion would decrease with the increase in water stress. Moreover, a recent study (Guo et al., 2016) suggested that high moisture conditions on the soil surface are conducive to the survival of fine roots, allowing a large amount of water and nutrients to be absorbed, and thereby increasing the root biomass on the soil surface. In this study, consistent with these findings, nearly half of the biomass was concentrated in the top layer of soil under all three water treatments. This was possibly also due to the fact that most organic matter and nutrients are stored close to the surface of the grassland soil (Hu et al., 2005), with fine roots in the upper layers playing a major role in nutrient absorption (Burton et al., 2000; Lynch, 2007). To meet the demands of plant growth, an increase in root biomass in this layer is therefore advantageous.

To summarize, the vertical and horizontal constraints on two soil habitats (shallow wide and deep narrow) did not constrain the overall biomass growth of the shallow-rooted grass, *L. perenne*. It indeed developed horizontal expansion at moderate drought in SW, although the overall expansion ability was limited under severe drought. However, *L. perenne* did not extend the vertical distribution of its root system into deep soil habitats, probably mainly because of the high water holding capacity of deep soil and the shallow rooting strategy of *L. perenne* itself. Together, these root responses of *L. perenne* will help our understanding of how herbaceous plants can adjust their belowground morphology to support their growth in harsh karst soil environments.

## Author Contributions

JL and JT designed the experiments. JZ, HS, and SL carried out the experiments supervised by JL and JT. JZ, and YZ analyzed experimental results supervised by JL and JT. JZ, JL, JW, JC, and JT wrote the manuscript.

## Conflict of Interest Statement

The authors declare that the research was conducted in the absence of any commercial or financial relationships that could be construed as a potential conflict of interest.

## Abbreviations

DN: deep narrow soil
FC: field water holding capacity
SRWC: soil relative water content
SW: shallow wide soil
SWC: soil water content.
